# Effect of Waste Ceramic Powder on the Properties of Alkali–Activated Slag and Fly Ash Pastes Exposed to High Temperature

**DOI:** 10.3390/polym13213797

**Published:** 2021-11-02

**Authors:** Gui-Yu Zhang, Sung-Chul Bae, Run-Sheng Lin, Xiao-Yong Wang

**Affiliations:** 1 Department of Architectural Engineering, College of Engineering, Kangwon National University, Chuncheon-si 24341, Korea; zhangguiyu@kangwon.ac.kr; 2 Department of Architectural Engineering, Hanyang University, 222, Wangsipri-ro, Sungdong-gu, Seoul 04899, Korea; sbae@hanyang.ac.kr; 3 Department of Integrated Energy and Infra System, College of Engineering, Kangwon National University, Chuncheon-si 24341, Korea; linrunsheng@kangwon.ac.kr

**Keywords:** waste ceramic powder, alkali-activated slag fly ash, high temperature, compressive strength, reaction product, meso-crack

## Abstract

This paper presents the effects of alkali-activated blast furnace slag and fly ash (AASF) paste added with waste ceramic powder (WCP) on mechanical properties, weight loss, mesoscopic cracks, reaction products, and microstructure when exposed to 300, 600, and 900 °C. Using waste ceramic powder to replace blast furnace slag and fly ash, the replacement rate was 0–20%. The samples cured at 45 °C for 28 days were heated to 300, 600, and 900 °C to determine the residual compressive strength and weight loss at the relevant temperature. We evaluated the deterioration of the paste at each temperature through mesoscopic images, ultrasonic pulse velocity (UPV), thermogravimetric analysis (TG), Fourier transform infrared (FTIR) spectroscopy, X-ray diffraction (XRD), and with a scanning electron microscope (SEM). Relevant experimental results show that: (1) with the increase in waste ceramic powder content, the compressive strength of samples at various temperatures increased, and at 300 °C, the compressive strength of all the samples reached the highest value; (2) the residual weight increased with the increase in the content of the waste ceramic powder; (3) with a further increase in temperature, all the samples produced more mesoscopic cracks; (4) at each temperature, with the rise in waste ceramic powder content, the value of the ultrasonic pulse velocity increased; (5) the TG results showed that, as the content of waste ceramic powder increased, the formation of C-A-S-H gel and hydrotalcite decreased; (6) XRD and FTIR spectra showed that, at 900 °C, the use of waste ceramic powder reduced the formation of harmful crystalline phases; (7) the SEM image showed that, at 900 °C, as the content of waste ceramic powder increased, the compactness of the sample was improved. In summary, the addition of waste ceramic powder can improve the mechanical properties of the alkali-activated paste at high temperatures, reduce the occurrence of cracks, and make the microstructure denser.

## 1. Introduction

Ordinary Portland Cement (OPC) is the most widely used cementitious material in architectural engineering. At present, the global annual output of OPC exceeds 4.1 billion tons [1,2]. In addition, carbon dioxide (CO_2_) emissions from cement production account for about 8% of global CO_2_ emissions [3,4], which has a large impact on global climate change [5]. In recent years, alternative materials that can reduce energy consumption and CO_2_ emissions have become the focus of research [6,7]. At present, alkali-activated materials have developed into promising alternatives to PC and have attracted the attention of many researchers [8,9]. The raw materials of alkali-activated materials are usually industrial byproducts such as blast furnace slag (BFS) and fly ash (FA). Their use not only significantly reduces CO_2_ emissions but also reduces environmental pollution. The chemical reaction of alkali solution with blast furnace slag and fly ash, the alkali-activated slag, and alkali-activated fly ash produced are the most straightforward and most extensive two. Xie [10] studied the performance of alkali-activated BFS and FA geopolymer recycled concrete. They believe that the combination of geopolymer binder and recycled concrete can exhibit excellent compression resistance. In addition, Xie [11] also studied the sulfate resistance of recycled aggregate concrete containing BFS and FA geopolymers. They found that the high BFS content of geopolymer recycled aggregate concrete has excellent sulfate resistance. The alkali activator promotes the dissolution of BFS and FA in solution. It then undergoes a series of dissociation, precipitation, and polymerization reactions to form a solid-phase calcium aluminosilicate hydrate (C-A-S-H) gel that can affect its mechanical properties and thermal durability [8,12,13].

At the same time, ceramic products have brought many conveniences to people’s lives due to their excellent performance, but discarded ceramic products have put tremendous pressure on landfills. Waste ceramics are produced during the polishing of the ceramic industry and the destruction of buildings [14,15]. At present, waste ceramics are often used to replace natural aggregates in concrete [16,17], which can improve the mechanical properties and durability of concrete [18,19,20]. Rashid [21] studied the effect of adding ceramic aggregates on the properties of concrete. The results show that adding ceramic aggregate can enhance the strength of concrete. However, this crude use of waste ceramics needs to be further improved. Li [22] found that waste ceramic powder can refine pores when added to concrete instead of cement. In addition, the activated alumina and silica in the waste ceramic powder dissolve in the pore solution and form a new calcium aluminosilicate hydrate (C–A–S–H) with the active ions (Ca^2+^ and OH^-^). The uniformly dispersed ceramic particles also shorten the transmission distance of ions from the active material to the nucleation site. These physical and chemical effects together affect the mechanical properties of materials. Kannan [23] found that concrete with waste ceramic powder has a higher strength and durability, mainly due to the filler effect of waste ceramic powder. Sun [24] reported that WCP can be used as a raw material for geopolymers. They pointed out that the compressive strength of geopolymers is affected by different alkali activators. When different alkali activators are used, the samples still have good thermal stability when heated to 1000 °C. Shohei [25] reported that WCP can be excited with NaOH and Na_2_SiO_3_ solutions. Their results showed that the ratio of alkaline solution/binder (L/B) will affect the fluidity of the mortar. In contrast, the density and compressive strength will be affected by the L/B ratio and curing temperature. In addition, their research found that mortar has the highest compressive strength when the L/B is 0.6, and the curing temperature is 90 °C. Koppert [26] used the industrial byproducts produced in brick calibration (surface polishing) to prepare red clay ceramic powder. They found that when waste red clay ceramic powder is used as a raw material for geopolymers, the mechanical properties of concrete are affected by the composition of the alkali activator and the CaO content in the red clay ceramic powder. Higher CaO content results in broader pore size distribution and higher porosity. Husein [27] studied the performance of alkali-activated concrete mixed with waste ceramic powder. They pointed out that the addition of waste ceramic powder can improve the working performance of concrete, and can enhance the durability of exposure to sulfuric acid.

Previously, many researchers have studied the high-temperature resistance of alkali-activated materials. Rashad [28] studied the influence of sodium silicate activator concentration on the thermal properties of alkali-activated slag. The research results show that the sodium silicate concentration is calculated as Na_2_O. Compared with the sample prepared with the Na_2_O concentration of 10.5%, the sample prepared with the Na_2_O concentration of 6.5% had higher residual strength at high temperatures. Cai and Ye [29] studied the alkali-activated slag exposed to high temperatures of 400, 600, and 800 °C. They pointed out that the addition of potassium ions inhibited the harmful crystallization and phase transition of the reaction phase at high temperatures and refined the pores. In other documents, the high-temperature resistance of alkali-activated slag added with WCP was studied. Huseien [14] studied alkali-activated slag and fly ash that was exposed to high temperatures of 27, 400, 700, and 900 °C with WCP added. They pointed out that the weight loss decreased with the addition of WCP. Rashad [15] studied the performance of alkali-excited slag with WCP when exposed to temperatures of 200, 400, 600, 800, and 1000 °C. They observed that after the alkali-activated slag sample added with WCP was heated, the compressive strength of the paste sample was improved. What is interesting is that the compressive strength of all samples is the lowest when the paste is exposed to 600 °C. Then the compressive strength increases with the increase in temperature, and the compressive strength is the highest at 1000 °C.

Although many studies were conducted on the addition of WCP to alkali-activated materials, the authors found minimal information about adding WCP to alkali-activated slag and fly ash at high temperatures. To fill the deficiencies in the literature, it is necessary to study the high-temperature resistance of the alkali-activated slag and fly ash paste added with WCP. Secondly, we used more experimental methods, such as increased mesoscopic image analysis and ultrasonic pulse velocity, compared with previous studies. In addition, the relationship between compressive strength and mesoscopic images is also analyzed. On the other hand, recycling waste ceramic powder to produce an alkali-activated paste not only protects the environment and reduces waste disposal, it is also beneficial in terms of high-temperature resistance and economy. Several experimental studies were conducted in this paper, including compressive strength, weight loss, mesoscopic image analysis, UPV, TG, FTIR, XRD, and SEM. Based on the results of these experimental studies, the relationship between the amount of WCP substitution and compressive strength, weight loss, mesoscopic image analysis, and ultrasonic pulse velocity was determined. We combined XRD and FTIR to analyze the phases of the net pulp at various temperatures. The correlation between compressive strength and ultrasonic pulse velocity, as well as the relationship between macroscopic properties, mesoscopic images, and microstructure were analyzed.

The main objectives of this paper are as follows: 1. Clarify the influence of high temperature on the mechanical properties of alkali-activated materials and the possibility of ultrasonic velocity as a non-destructive testing method for compressive strength; 2. Analyze the microscopic characteristics of the product composition and morphology at each temperature; 3. Study the development of micro-cracks at each temperature, and analyze the corresponding relationship between the macro-micro-micro scale of the material properties.

## 2. Materials and Methods

### 2.1. Raw Materials and Alkali Activator

**Figure 1:**
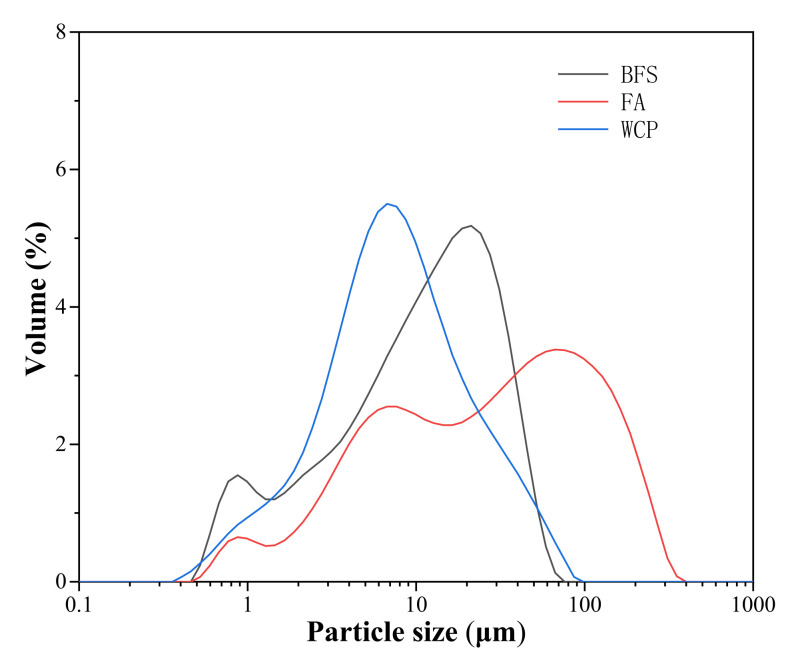
Particle size distribution of BFS, FA, and WCP.

**Figure 2:**
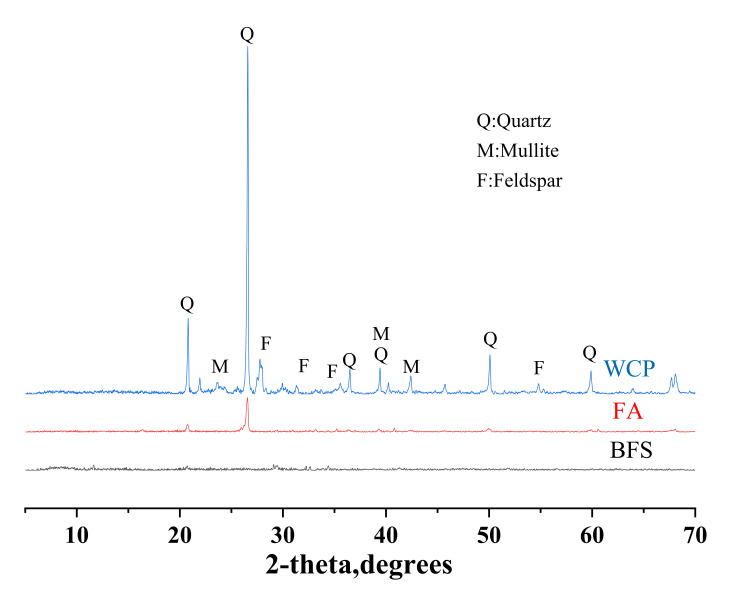
XRD patterns of BFS, FA, and WCP.

In this study, BFS was supplied by South Korea’s Asia Cement Company (Seoul, Korea). Power plants collect class F fly ash (Seoul, Korea). Waste ceramic powder is from the ceramic tiles ordered in building destruction (Seoul, Korea), which a ball mill grinds. Table 1 lists the chemical composition and ignition loss of blast furnace slag, fly ash, and waste ceramic powder, determined by using X-ray fluorescence (XRF) analysis. As shown in Table 1, the main oxide components of blast furnace slag are CaO, SiO_2_, and Al_2_O_3_, and the main oxide components of fly ash and waste ceramic powder are SiO_2_ and Al_2_O_3_. The loss on ignition of blast furnace slag, fly ash, and waste ceramic powder measured by XRF were 1.25, 3.92, and 1.19%, respectively. The particle size distribution (PSD) of blast furnace slag, fly ash, and waste ceramic powder is shown in Figure 1. The XRD patterns of blast furnace slag, fly ash, and waste ceramic powder are shown in Figure 2. The peaks of silica, mullite, and feldspar can be found in the waste ceramic powder. Silica is the main crystalline phase of fly ash. The XRD pattern of blast furnace slag does not have any sharp peaks due to the material’s amorphous characteristics [30]. Table 2 lists the median particle size (d50) and specific gravity of blast furnace slag, fly ash, and waste ceramic powder. PSD measures the median particle size (d50), and the specific gravity is determined according to ASTM C188 [31].

In this study, two types of alkaline solutions were mixed to activate the reactivity of the mixture: 98% sodium hydroxide particles (GR grade); and liquid sodium silicate (water-glass: Na_2_O = 9.5%, SiO_2_ = 29% and water = 61.5%). We dissolved the sodium hydroxide particles in water to obtain a sodium hydroxide solution (NH solution), then left the solution for one day to cool down, and then added ions; the molar ratio of SiO_2_:Na_2_O was 1.2 [27].

### 2.2. Mixing Ratio and Sample Preparation

Table 3 lists the detailed mixing ratios for blast furnace slag, fly ash, and waste ceramic powder in the prepared paste. The weight percentages of waste ceramic powder were 0, 10, and 20%, respectively. To ensure the mechanical properties of the mixed paste, the total replacement rate of ceramic waste powder should not exceed 20%. The names representing the above virgin pulp were WCP-0, WCP-10, and WCP-20. A fixed alkali solution/binder ratio of 0.5 and a water/binder ratio of 0.4064 were used to prepare the samples. For all paste, the mixed alkali activator accounted for 4 wt% of the binder mass based on Na_2_O, and remained unchanged [32].

We weighed all the materials in proportion to the quality and used a stirrer to prepare the paste, then poured it into the cube and cuboid molds with dimensions of 50 × 50 × 50 mm and 40 × 40 × 160 mm. We wrapped the paste sample with a film to prevent the loss of alkali solution, and then put it in a 45 ± 2 °C curing chamber for curing [15]. The current curing condition of 45 °C is feasible for prefabricated elements. Regarding this material used on the construction site, more further studies are necessary.

After one day, the mold was removed, the sample was sealed with a film and cured in the curing chamber. To evaluate the high-temperature resistance, the samples cured for 28 days were placed in a muffle furnace at ambient temperature and heated to 300, 600, and 900 °C at a fixed heating rate (5 °C/min). The sample remained at the target temperature for 2 h to ensure that the temperature distribution of the entire sample was uniform and the sample was naturally cooled to ambient temperature in the muffle furnace.

### 2.3. Test Method

#### 2.3.1. Compressive Strength and Sample Weight

According to the ASTM C109 standard for the compressive strength test, three samples of each paste before and after heating were tested to determine the average value [33].

#### 2.3.2. Weight Changes before and after Heating

After heating with a muffle furnace, we carefully took out the sample, and used an electronic scale to measure the weight of the sample before and after heating. The weight of the same sample under different heating temperatures was measured, and the average value of three samples was measured.

#### 2.3.3. Mesoscopic Image Analysis

We observed the mesoscopic images of the sample before and after heating with a microscope (LEICA Z16 APO, Leica, Germany), with a magnification of 76.

#### 2.3.4. Ultrasonic Pulse Velocity

The UPV test used a sample with a size of 40 × 40 × 160 mm. According to the ASTM C597 standard, a portable ultrasonic tester (Pundit Lab, Proceq company, Switzerland) was used for nondestructive UPV testing of samples before and after heating and measurements from three samples were used to find the average value.

#### 2.3.5. Thermogravimetric Analysis

Thermogravimetric analysis (SDT Q600, TA Instruments, USA) was used to characterize the samples cured at each temperature for 28 days to analyze the dehydration and thermal decomposition processes of the reaction phase [34,35]. About 30 mg of powder was placed in the ceramic crucible of the thermogravimetric analyzer and heated from 20 to 1000 °C at a heating rate of 15 °C/min.

#### 2.3.6. Fourier Transform Infrared Spectroscopy

A spectrometer (Frontier, PerkinElmer) with a resolution of 0.4 cm^−^1 was used to test the sample. Before each measurement, a background scan of the ZnSe diamond crystal was performed. Each sample was scanned 16 times in the range of 450 to 4000 cm^−1^.

#### 2.3.7. X-ray diffraction

An X-ray diffractometer (X’Pert PRO MPD diffractometer, Panalytical, Almelo, The Netherlands) was used to scan the sample after curing for 28 days and heating. We scanned the sample from 5 to 50° with a step size of 0.013° (2θ), and the cumulative scan time was 8.67 s [36].

#### 2.3.8. Scanning Electron Microscope

We used an ultra-high resolution scanning electron microscope (UHR-SEM, S-4800, Hitachi) to observe the samples microscopically and selected the flake samples for testing. The accelerating voltage was 15 kv, and the emission current was 7000 nA. Prior to microscopic observation, the surfaces of the samples were coated with platinum using an ion-sputter coater. 

## 3. Results

### 3.1. Compressive Strength and Weight Changes before and after Heating

**Figure 3:**
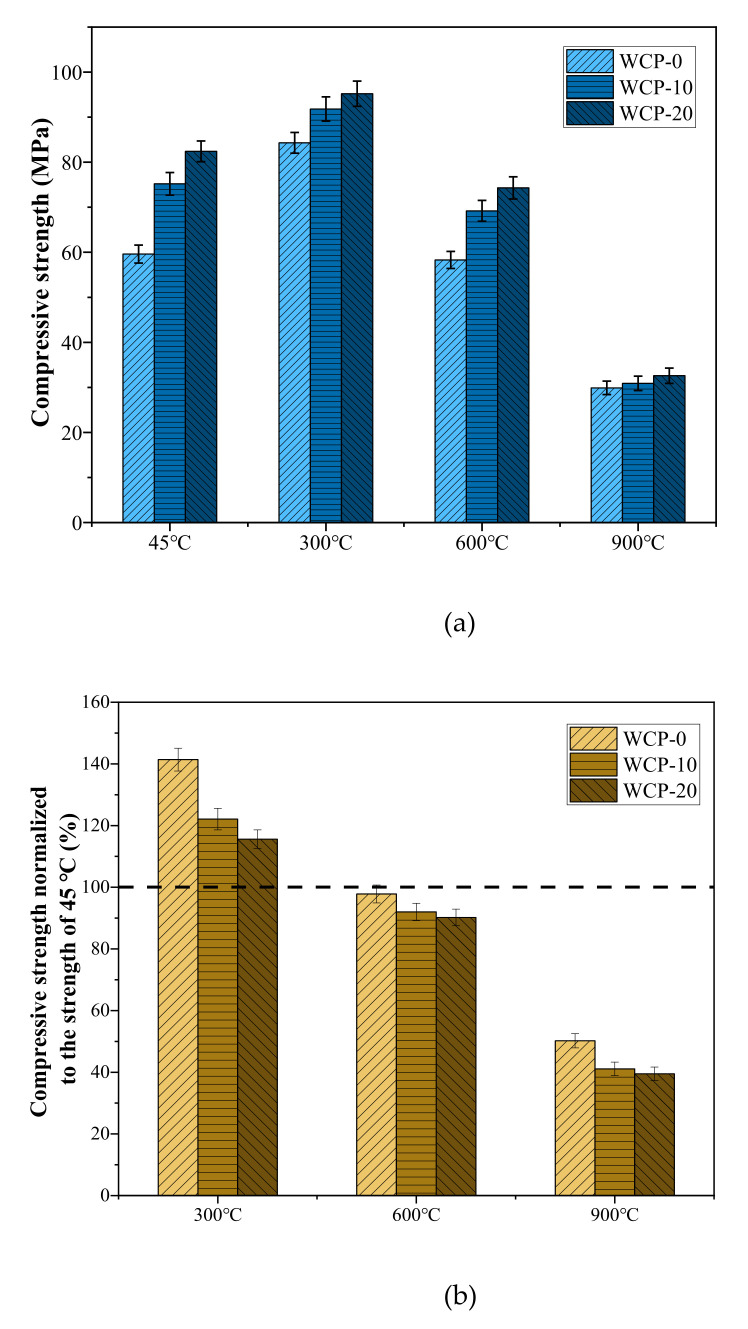
(**a**) Effect of the WCP amount on the compressive strength of pastes after exposure to elevated temperatures. (**b**) Compressive strengths of sample normalized to the strength of 45 °C.

By measuring the compressive strength of the samples before and after heating, the influence of the replacement amount of waste ceramic powder on the mechanical properties of the paste was evaluated. Figure 3a shows the compressive strength of all samples at 45, 300, 600, and 900 °C after curing for 28 days. The WCP-20 sample showed the highest compressive strength at all temperatures. Figure 3b shows the compressive strength of the sample after normalization at 45 °C. When the samples were processed at 300 °C, the compressive strength of all samples increased, especially WCP-0, which had the highest increase in rate of compressive strength. Subsequently, the compressive strength decreased with increasing temperature.

For the samples cured at 45 °C for 28 days, as the content of waste ceramic powder increased from 0 to 10 and 20%, the compressive strength was 59.6, 75.2, and 82.4 MPa, respectively. This is because the replacement amount of waste ceramic powder increases, which increases the percentage of silica, which is conducive to the formation of C-A-S-H gel with a high Si/Al ratio [37]. The presence of silicon dioxide enhances the reaction process and introduces more silicon into the polymer chain, contributing to the increase in strength.

At 300 °C, all samples had excellent mechanical properties. Their compressive strengths were all recorded at the highest value, respectively, 84.3, 91.8, and 95.2 MPa. After heating to 300 °C, the increase in compressive strength may be related to the further reaction of unreacted blast furnace slag, fly ash, and waste ceramic powder. At 300 degrees, the humidity in the surface zone and internal zone of specimens may be different. At the surface zone, the humidity is very low; further reactions are difficult to occur. However, at the internal zone, the so-called internal autoclaving effect will also lead to an increase in strength [38]. Above 100 °C, residual alkaline solution in alkali-activated pastes can be liberated as a form of steam, affecting the surrounding phases of alkali-activated paste. Alkaline steam cannot be released from the sample fast enough, creating a state of high pressure inside the paste, mainly due to insufficiently connected porosity [38]. Summarily, the internal autoclaving effect in the internal zone can overwhelm the low humidity effect in the surface zone. The overall result is an increase in strength. In addition, as the content of waste ceramic powder increased, the increase in compressive strength decreased. It may be that the presence of waste ceramic powder reduces the further reaction of the blast furnace slag.

When the temperature increased from 300 to 600 °C, the compressive strength of all samples decreased, but compared with the sample at 45 °C, the decrease in compressive strength was not significant. The reason for the decrease in compressive strength at this stage is related to the decomposition of C-A-S-H gel (see Section 3.7). As the temperature further increased to 900 °C, the compressive strength dropped sharply. This may be related to the crystallization and phase transition of the sample (see Section 3.7).

### 3.2. Macroscopic Weight Change

**Figure 4:**
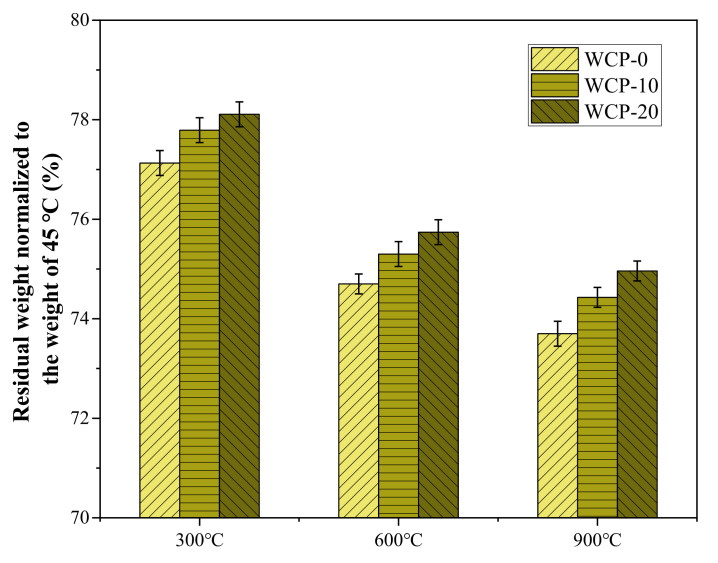
Residual weight of sample normalized to the weight of 45 °C.

Figure 4 shows residual weight percentages of WCP-0, WCP-10, and WCP-20 samples at different temperatures. There was a proportional relationship between the residual weight and the waste ceramic powder content. As the temperature increased, the weight of the sample decreased. This is due to the dehydration and decomposition of the C-A-S-H gel. At 900 °C, when the content of waste ceramic powder increased from 0% to 10% and 20%, the residual weight was 73.7%, 73.43%, and 74.96%, respectively. This shows that in the samples containing waste ceramic powder, as the content of waste ceramic powder increased, the thermal compatibility of the sample increased and the degree of deterioration decreased. The literature [14] also observed similar results. As the content of waste ceramic powder increases, the quality loss decreases.

### 3.3. Mesoscopic Image Analysis

**Figure 5:**
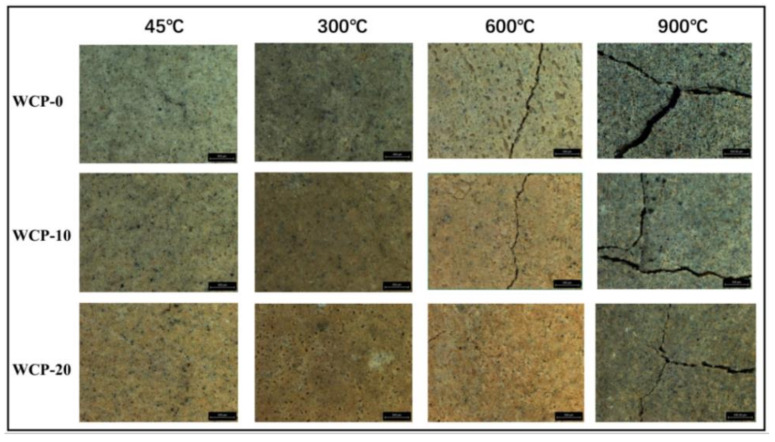
Mesoscopic images of all samples at different temperatures at 76 magnification.

When the samples were exposed to different temperatures, the physical and chemical properties of the samples changed due to heat, and the samples showed different colors and cracks occurred. Figure 5 shows the mesoscopic images of WCP-0, WCP-10, and WCP-20 treated at different temperatures for 2 hours. At 45 °C, when the waste ceramic powder was added to the alkali-excited slag fly ash, the color of the sample became yellow with the color of the waste ceramic powder. At 300 °C, the shape of the sample was complete, but there were some microcracks. This may be related to the dehydration of C-A-S-H [38]. When the temperature rose to 600 °C, visible cracks appeared on the surface of the sample. The difference from the observation results at different temperatures was that the crack gap became smaller as the replacement amount of waste ceramic powder increased. Then, when the temperature was further heated to 900 °C, large cracks visible to the naked eye appeared on the sample’s surface. Compared with the sample at 600 °C, the crack gap was further enlarged. This means that the thermal degradation of the sample is severe at 900 °C. In addition, no explosive peeling of the sample was observed at any temperature.

### 3.4. Ultrasonic Pulse Velocity (UPV)

**Figure 6:**
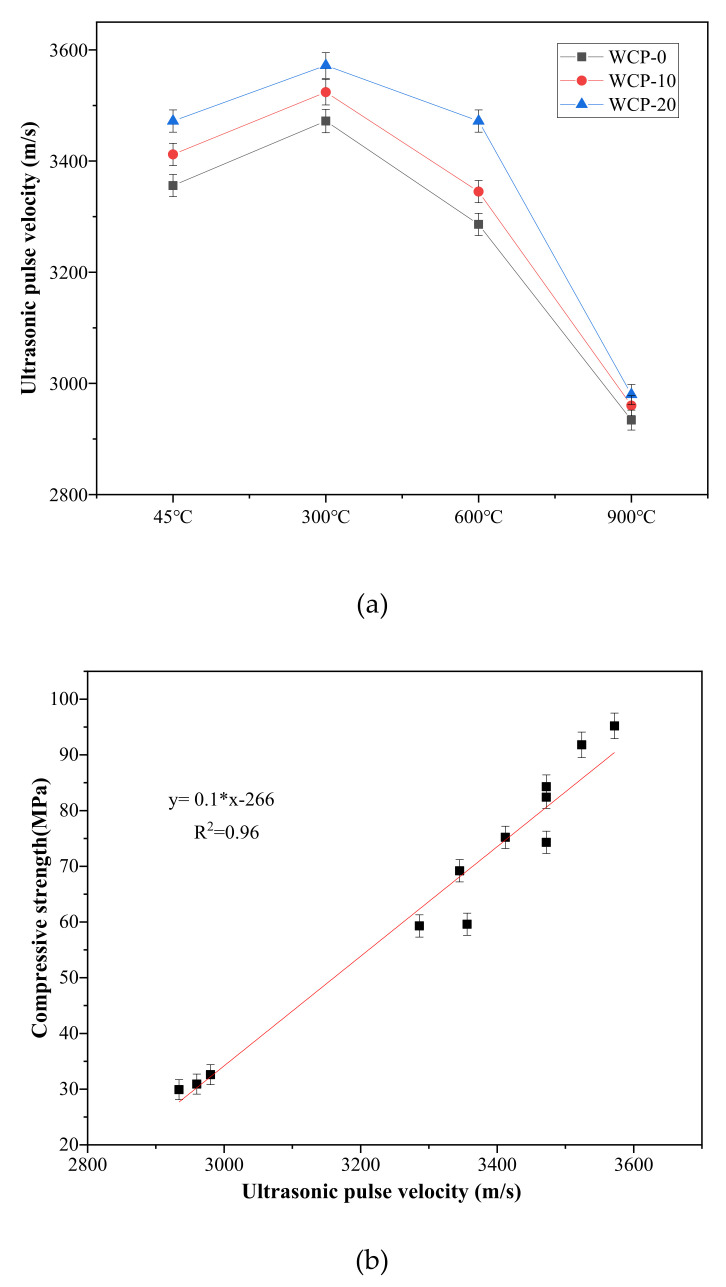
(**a**) UPV of mixed pastes at 45, 300, 600 and 900 °C; (**b**) The relationship between compressive strength and UPV.

As far as cement concrete is concerned, UPV is affected by w/b, curing age, admixtures, water, and aggregates [39,40]. For the samples under high temperature in this study, Figure 6a shows the UPV results of WCP-0, WCP-10, and WCP-20 samples at various temperatures. The UPV values of WCP-0, WCP-10, and WCP-20 samples all reached the highest at 300 °C, which is related to the further reaction of unreacted blast furnace slag, fly ash, and waste ceramic powder. After 300 °C, the UPV value decreased with the increase in temperature, which indicated that the deterioration of all samples increased with the rise in temperature. The cause of the decrease in UPV may be related to the decomposition of C–A–S–H gel. After 300 °C, the UPV value change trend was consistent with the UPV value change of cement at high temperatures [41]. Compared with the control group (WCP-0) at each temperature, the UPV value of WCP-20 was higher. This is because waste ceramic powder introduces more silica, which enhances the geopolymerization process and contributes to the geopolymer chain. An introduction of more silicon contributes to the increase in strength [37].

Figure 6b shows the relationship between UPV and compressive strength [42]. It can be seen that they have a very strong linear relationship, y = 0.1 × − 266, and the coefficient of determination is R^2^ = 0.96. The standard error of the slope is 0.0057, and the standard error of the intercept is 19. In addition, when testing UPV, there are also differences between different samples due to the unevenness of the samples. When processing experimental data, error bars are added (shown in Figure 6a,b).

### 3.5. Thermogravimetric Analysis

**Figure 7:**
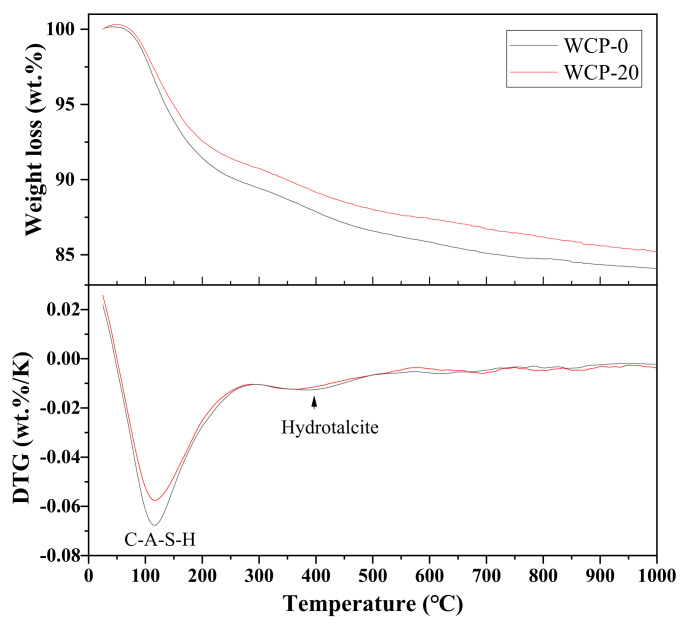
TG-DTG curves of pastes.

Figure 7 shows the thermogravimetric and differential thermogravimetric curves (TG-DTG) of the alkali-excited paste sample cured for 28 days. The TG-DTG curve used in this study was heated from room temperature to 1000 °C. It can be seen from the figure that the mass loss of all samples is faster before 110 °C, which is mainly due to the loss of free water in the samples [43]. For the temperature range corresponding to the mass loss of the reaction product after heating, please refer to the report in [44]. The central peak of DTG was about 110–130 °C, which was mainly the dehydration of C–A–S–H gel [44]. Compared with the control group WCP-0, the peak value of WCP-20 was smaller, which indicates that the reactivity of the ceramic powder was low, and the amount of C-A-S-H gel produced was reduced. The soft peak at 330–440 °C in DTG can be attributed to the decomposition of the hydrotalcite phase [29,44,45]. In addition, the peak intensity of the hydrotalcite phase of the sample WCP-20 was slightly weakened, which may be caused by the lower Mg content in the waste ceramic powder. With the increase in temperature, the mass loss of all samples increased, but the mass loss decreased with the rise in the content of the waste ceramic powder. This is consistent with the macroscopic quality loss measurement results. Compared with WCP-0 without waste ceramic powder, the WCP-20 sample containing waste ceramic powder had better thermal stability [15].

### 3.6. Fourier Transform Infrared Spectroscopy

**Figure 8:**
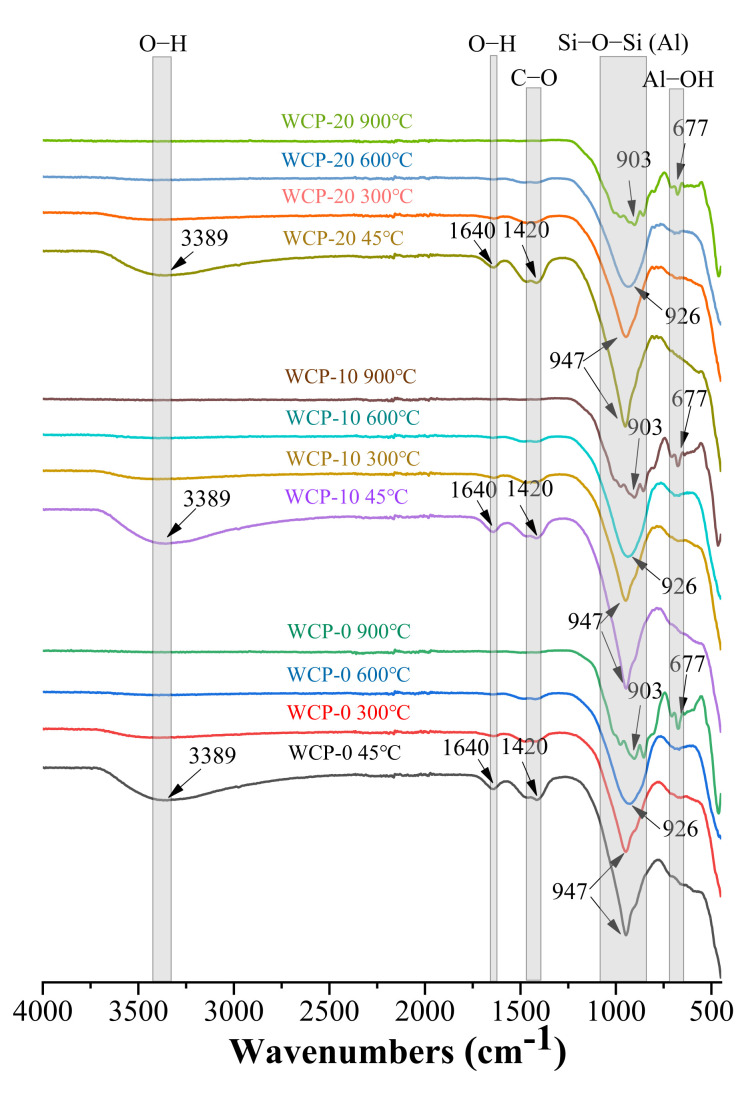
FTIR spectra of all samples before and after heating.

Figure 8 shows the FTIR spectra of all samples at various temperatures after 28 days of curing. The tensile vibration peak of the O-H bond was detected near the wavenumber of 3392 cm^−1^, while the bending vibration peak of the O-H bond appeared near the wavenumber of 1640 cm^−1^ [46]. When the sample was heated to 300 °C, the tensile vibration peak and the bending vibration peak corresponding to the O-H bond almost disappeared, indicating extensive dehydroxylation in each phase, including C-A-S-H. At 45 °C, the C–O bond absorption peak was observed near 1420 cm^−1^, and the intensity of the C–O bond absorption peak gradually attenuated as the temperature increased and disappeared at 900 °C. This shows that alkali-activated slag and waste ceramic powder samples are prone to carbonization (sodium bicarbonate and calcium carbonate or a blend of the two), and carbonates are decomposed at high temperatures. The peaks appearing in the range of 997–938 cm^−1^ wavenumber correspond to the asymmetric stretching vibration of the Si-O-Si (Al) bond in the C-A-S-H gel [47,48,49]. As the temperature increased from 300 to 900 °C, the intensity of the Si–O–Si (Al) bond absorption peak weakened and shifted to a lower wavenumber. This phenomenon indicates that the aluminosilicate network structure of the C-A-S-H gel has changed, which may be the cause of the degradation of the mechanical properties of the paste [29].

### 3.7. X-ray Diffraction

**Figure 9:**
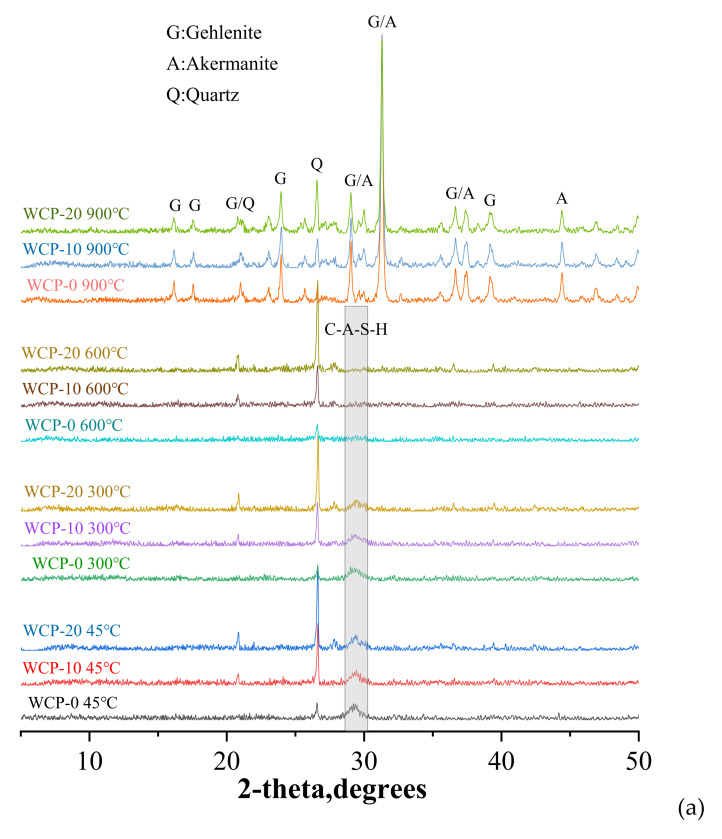

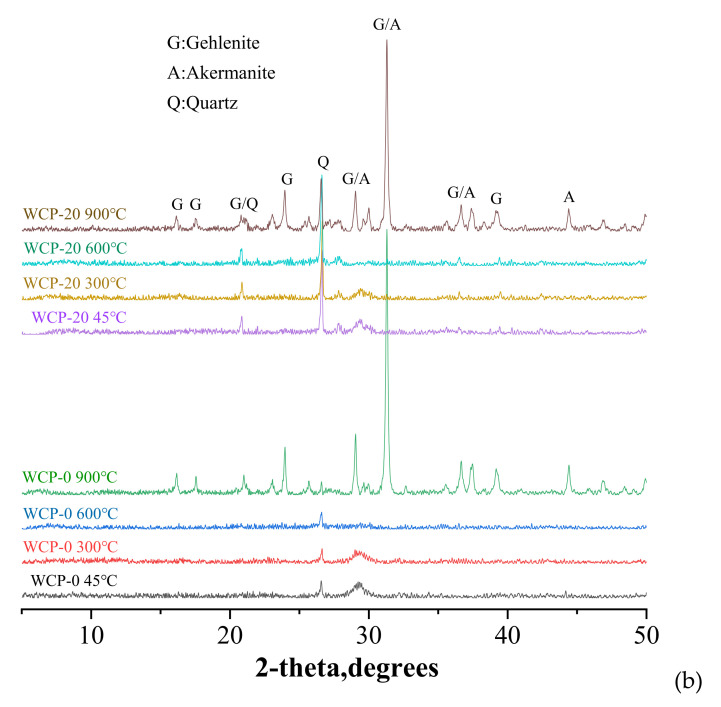
(**a**) XRD patterns of all the samples before and after heating. (**b**) XRD patterns of WCP-0 and WCP-20 before and after heating.

Figure 9a shows the XRD spectra of all the samples exposed to various temperatures. The peak of quartz was between 26° and 27°. In waste ceramic powder and fly ash, quartz always exists due to its inert reaction. At the same time, as the content of waste ceramic powder increased, the intensity of the quartz peak also increased. In addition, a small amount of feldspar was also observed in the samples containing waste ceramic powder.

In the heating test, the paste sample underwent decomposition and transformation of the reaction phase. As the heating temperature increased from 45 to 600 °C, the C-A-S-H semi-crystalline phase of all samples decreased sharply. Compared with the sample before heating, the hump was more dispersed, and it was almost unrecognizable at 600 °C, which means the gradual dehydration and decomposition of the C-A-S-H semi-crystalline phase [50]. References [15,29] also have similar findings. After the sample was heated at 900 °C, the shape and number of peaks changed radically, which means that the sample changed from an amorphous phase and a semi-crystalline phase to a crystalline phase, accompanied by the formation of new substances.

Gehlenite (Ca_2_Al_2_SiO_7_), Akermanite (Ca_2_MgSi_2_O_7_), and Quartz (SiO_2_) crystalline phases were detected in samples exposed to 900 °C. Gehlenite and Akermanite crystalline phase peaks appeared in the original C-A-S-H semi-crystalline phase hump. The main crystalline phase, Akermanite, crystallized at 710 °C (983 K) [51]. The crystalline phase of Gehlenite was formed at a temperature of 697 °C (970 K) [52]. These crystalline phases may lead to the transformation of the void structure from micro to meso or even macro size [29,53].

Figure 9b shows the XRD patterns of samples WCP-0 and WCP-20 at various temperatures. As the temperature increased from 600 to 900 °C, the peaks of Gehlenite and Akermanite crystal phases appeared. The results of Section 3.1 compressive strength show that when the temperature increased from 600 to 900 °C, the compressive strength dropped sharply, which shows that the formation of Gehlenite and Akermanite crystalline phases is unfavorable [27]. For all samples heated at 900 °C, combined with the analysis of Figure 9a,b, the peak intensity of the crystalline phase weakened slightly as the replacement amount of the waste ceramic powder increased. This finding indicates that the addition of waste ceramic powder reduces the crystallization and phase transition of the WCP-20 sample at 900 °C.

### 3.8. Scanning Electron Microscope

**Figure 10:**
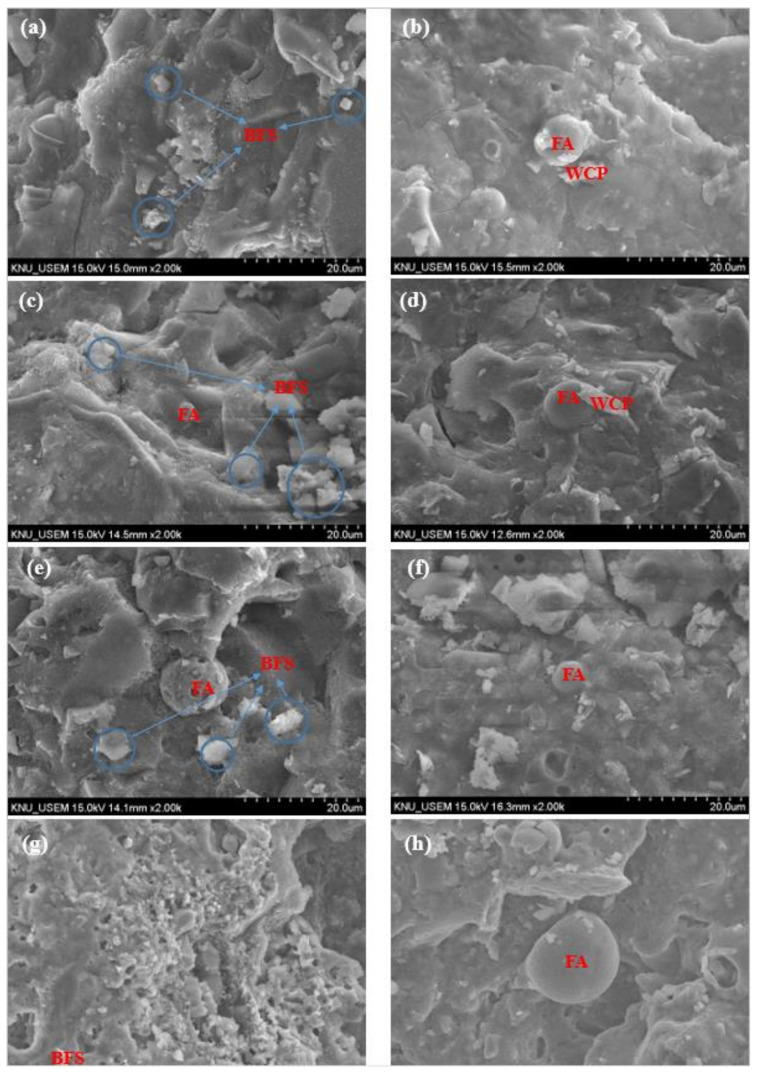
SEM images of WCP-0 and WCP-20 magnified 2000 times before and after heating. (**a**) WCP-0, 45 °C; (**b**) WCP-20, 45 °C; (**c**) WCP-0, 300 °C; (**d**) WCP-20, 300 °C; (**e**) WCP-0, 600 °C; (**f**) WCP-20, 600 °C; (**g**) WCP-0, 900 ℃; (**h**) WCP-20, 900 °C.

Figure 10 shows the SEM images of WCP-0 and WCP-20 samples after exposure to 45, 300, 600, and 900 °C. Unreacted slag particles (irregular jagged) could be observed at all temperatures. In addition, some spherical particles of fly ash were also visible. Fly ash contains a large number of particles with hollow spheres. When these hollow spheres are partially dissolved, they will form highly dispersed pores in the matrix [54]. Although there are some angular and irregular waste ceramic powder particles in the WCP-20 sample, replacing blast furnace slag and fly ash with 20% waste ceramic powder results in a more compact and uniform microstructure. The incorporation of waste ceramic powder can improve the compactness of the paste, and the waste ceramic powder can fill the voids [15]. When the sample was heated to 900 °C, the morphology of the microstructure underwent a fundamental change, and the original compact structure became looser. At the same time, more holes appeared at 900 °C. The formation of crystalline phases may cause this after further decomposition of C–A–S–H as the temperature increases. The literature [51] also found a similar phenomenon. This caused more microcracks in the mesoscopic view, which led to a significant reduction in the compressive strength in the macroscopic view.

## 4. Discussion

The third part analyzed the results of each experiment. This section will discuss the correlation of the results at different temperatures. The correlation is discussed as follows:

### 4.1. The Temperature Range of 45–300 °C

According to the macro study, WCP-20 samples with waste ceramic powder showed the highest compressive strength at all temperatures; all samples increased in compressive strength at 45–300 °C, but with the increase in waste ceramic powder content, the rate of growth in compressive strength decreased. Although the samples caused dehydration of the C-A-S-H gel at this stage, the compressive strength of all samples was increased compared with that at 4 °C. The reason for the increase in compressive strength may be related to the further reaction of unreacted blast furnace slag, fly ash, and ceramic powder. According to the results of the mesoscopic images, from 45 to 300 °C, microcracks appeared on the surface of the sample. In other words, from 45 to 300 °C, the macroscopic properties and mesoscopic analysis research were inconsistent. This is due to the further reaction of unreacted furnace slag, fly ash, and ceramic powder. The mesoscopic image was not sufficient for detecting the formation of new hydration products. However, the presence of microcracks can enhance the connectivity of the matrix’s void network, which is conducive to the escape of alkaline solution [29]. In addition, the increase in temperature is consistent with the development of mesoscopic cracks [55].

### 4.2. The Temperature Range of 300–600 °C

When exposed to 600 °C, as the content of waste ceramic powder increased, microcracks decreased and crack width decreased. The decrease in compressive strength is attributed to the decomposition of the C-A-S-H gel. According to FTIR results, temperatures as high as 600°C accelerate the breaking process of chemical bonds in the alkali-activated matrix. This is consistent with the results of the literature [56]. At the same time, XRD results found that the humps of C–A–S–H became more dispersed at 600 °C and were almost unrecognizable. In addition, samples with high calcium content led to a high degree of damage, a significant loss of strength, and an increase in volume, leading to crack development [14,57].

### 4.3. The Temperature Range of 600–900 °C

According to the results of mesoscopic image observation and macroscopic research, the compressive strength decreased at 600–900 °C, the crack width was further enlarged, the surface became uneven, and the compressive strength of all samples dropped sharply. According to the results of XRD, the new crystalline phase Gehlenite (Ca_2_Al_2_SiO_7_) and Akermanite (Ca_2_MgSi_2_O_7_) were detected in the sample at 900 °C, and the formation and phase transformation of harmful crystalline phases were reduced with the increase in awaste ceramic powder content. At the same time, according to FTIR results, the strength of the Si–O–Si (Al) bond weakened and shifted to a lower wavenumber, indicating that the chemical bond in the alkali-activated matrix was broken and reorganized. In addition, after heating to 900 °C, SEM observed that, compared with the WCP-0 sample without waste ceramic powder, the looseness of the WCP-20 sample containing waste ceramic powder was reduced. These findings indicate that it is beneficial to add waste ceramic powder when the sample is exposed to 900 °C.

## 5. Conclusions

This paper introduced the experimental research results of the compressive strength, weight loss, mesoscopic image analysis, and microstructure of the alkali-activated slag and fly ash samples with waste ceramic powder added before and after heating. We combined XRD and FTIR to perform phase analysis on all samples at various temperatures before and after heating. According to various experimental results, the following conclusions are drawn:

The compressive strength results show that as the content of waste ceramic powder increases, the compressive strength increases. When exposed to 300 °C, the compressive strength of all samples reached the highest value. 

As the temperature increases, the weight of the sample decreases, which is caused by the dehydration and decomposition of C-A-S-H. In addition, as the content of waste ceramic powder increases, the residual weight increases. 

When heated to 300 °C, the color of all the samples darkened slightly, and slight cracks formed. When heated to 900 °C, all samples showed large cracks visible to the naked eye.

As the content of waste ceramic powder increases, the value of ultrasonic pulse velocity increases. This may be that the addition of waste ceramic powder introduces more active silica, enhances the geopolymerization process and introduces more silicon in the geopolymer chain, which contributes to the improvement of strength and the value of UPV.

The analysis of TG results showed that the formation of C-A-S-H gel in sample WCP20 was reduced compared with the control group WCP0. In addition, a decrease in the formation of hydrotalcite was observed at 330–440 °C. 

According to the analysis results of FTIR, as the temperature increases from 300 °C to 900°C, the intensity of the Si–O–Si (Al) bond absorption peak weakens and shifts to a lower wavenumber. This means that the chemical bonds are broken and reorganized. 

The analysis of XRD results shows that a new peak appears after the sample is heated at 900°C, which is related to the formation of a crystalline phase. With the increase in WCP content, the peak intensity of the crystalline phase decreases, which indicates that the addition of WCP reduces the formation of harmful crystalline phases.

According to the SEM observation results, compared with the control group WCP-0 without waste ceramic powder, the microstructure of the sample WCP-20 containing waste ceramic powder became more compact and uniform. When the sample was heated to 900 °C, the morphology of the microstructure underwent a fundamental change.

This article introduces a preliminary research result. The alkali-activated materials with added waste ceramic powder need further research as follows: 1. The influence of curing temperature on the high-temperature resistance of alkali-activated materials; 2. The effect of different activators on the high-temperature resistance of alkali-activated materials; 3. The influence of different mixing ratios of waste ceramic powder, blast furnace slag and fly ash on the high-temperature resistance of alkali-activated materials.

## Figures and Tables

**Table 1:** Chemical properties of BFS, FA, and WCP used in this study.

Type of Binder	Chemical Compositions (wt.%)									LOI ^1^
	CaO	SiO_2_	Al_2_O_3_	Fe_2_O_3_	MgO	SO_3_	K_2_O	Na_2_O	ZnO	
BFS	38.9	32.2	15.7	0.65	7.08	2.65	0.61	0.30	-	1.25
FA	9.97	50.2	20.2	7.68	3.13	0.30	1.53	0.92	-	3.92
WCP	9.32	66.1	15.9	2.38	0.58	0.42	1.93	1.12	0.15	1.19

^1^ loss on ignition.

**Table 2:** Physical properties of BFS, FA and WCP.

	BFS	FA	WCP
Particle size d50 (μm)	12.2	30.2	7.92
Specific gravity	2.82	2.28	2.61

**Table 3:** Mixtures of paste samples.

Specimen	Binder			Alkali-Activator		Outside Water	Water in Water-Glass	Water/Binder	Alkali Solution/Binder
	BFS	FA	WCP	NaOH	Water-Glass				
WCP-0	80	20	0						
WCP-10	72	18	10	3.2	16	30.8	9.84	0.4064	0.5
WCP-20	64	16	20						

## Data Availability

The data presented in this study are available on request from the corresponding author.
